# SIX1 cooperates with RUNX1 and SMAD4 in cell fate commitment of Müllerian duct epithelium

**DOI:** 10.1038/s41418-020-0579-z

**Published:** 2020-06-22

**Authors:** Jumpei Terakawa, Vanida A. Serna, Devi M. Nair, Shigeru Sato, Kiyoshi Kawakami, Sally Radovick, Pascal Maire, Takeshi Kurita

**Affiliations:** 1 Department of Cancer Biology and Genetics, The Comprehensive Cancer Center, The Ohio State University, Columbus, OH USA; 2 Division of Biology, Center for Molecular Medicine, Jichi Medical University, Shimotsuke, Tochigi Japan; 3 Department of Pediatrics, Rutgers Robert Wood Johnson Medical School, Rutgers Biomedical and Health Sciences, New Brunswick, NJ USA; 4 Institut Cochin, INSERM U1016, CNRS UMR 8104, Université Paris Descartes, Paris, France; 5 Present Address: Division of Transgenic Animal Science, Advanced Science Research Center, Kanazawa University, Kanazawa, Japan

**Keywords:** Development, Gene expression, Molecular biology, Reproductive disorders

## Abstract

During female mammal reproductive tract development, epithelial cells of the lower Müllerian duct are committed to become stratified squamous epithelium of the vagina and ectocervix, when the expression of ΔNp63 transcription factor is induced by mesenchymal cells. The absence of ΔNp63 expression leads to adenosis, the putative precursor of vaginal adenocarcinoma. Our previous studies with genetically engineered mouse models have established that fibroblast growth factor (FGF)/mitogen-activated protein kinase (MAPK), bone morphogenetic protein (BMP)/SMAD, and activin A/runt-related transcription factor 1 (RUNX1) signaling pathways are independently required for ΔNp63 expression in Müllerian duct epithelium (MDE). Here, we report that sine oculis homeobox homolog 1 (SIX1) plays a critical role in the activation of ΔNp63 locus in MDE as a downstream transcription factor of mesenchymal signals. In the developing mouse reproductive tract, SIX1 expression was restricted to MDE within the future cervix and vagina. SIX1 expression was totally absent in SMAD4 null MDE and was reduced in RUNX1 null and FGFR2 null MDE, indicating that SIX1 is under the control of vaginal mesenchymal factors: BMP4, activin A and FGF7/10. Furthermore, *Six1*, *Runx1*, and *Smad4* gene-dose-dependently activated ΔNp63 expression in MDE within the vaginal fornix. Using a mouse model of diethylstilbestrol (DES)-associated vaginal adenosis, we found DES action through epithelial estrogen receptor α (ESR1) inhibits activation of ΔNp63 locus in MDE by transcriptionally repressing SIX1 and RUNX1 in the vaginal fornix.

## Introduction

In mammals, the majority of the female reproductive tract (FRT) develops from the Müllerian ducts (MDs) [1–6]. During embryogenesis, the MDs undergo a dynamic transformation from simple tubes consisting of homogeneous epithelium and mesenchyme into distinct organs, namely the oviduct, uterus, cervix, and vagina [7, 8]. Classic tissue recombination studies have established that organ-specific mesenchyme induces differentiation of MD epithelium (MDE) into epithelia with unique morphology and functions [9–11]. In the lower MD, epithelial cells are committed to become stratified squamous epithelium of ectocervix and vagina (together referred to as “vagina” hereafter), as the expression of ΔNp63 transcription factor is induced by vaginal mesenchyme [12–14]. In MDE of the developing vagina, the expression of ΔNp63 is activated by mesenchymal paracrine factors: bone morphogenetic protein (BMP) 4, activin A (ActA), and fibroblast growth factor (FGF) 7 or 10 [15, 16]. SMAD4 is essential for the activation of ΔNp63 in MDE, and this transcription factor binds on the 5′ sequence adjacent to the transcription start site (TSS) of ΔNp63 in future vaginal epithelium (VgE) [16]. This SMAD-dependent activation of the ΔNp63 locus requires runt-related transcription factor 1 (RUNX1). In MDE, the expression of RUNX1 is activated by ActA through a SMAD-independent mechanism [15]. In addition, activation of the mitogen-activated protein kinase (MAPK) pathway by FGF7/10-FGF receptor 2 IIIb (FGFR2IIIb) is essential for the activation of the ΔNp63 locus in MDE [15]. Once the ΔNp63 locus is activated in MDE, the transcriptional activity of the ΔNp63 locus is maintained by ΔNp63 protein itself independent of mesenchymal factors [11, 12, 16].

BMP4-SMADs, ActA-RUNX1, and FGF7/10-MAPK pathways are independently required for the vaginal cell fate commitment of MDE. Inactivation of *Smad4*, *Runx1*, or *Fgfr2* in MDE results in uterine epithelial differentiation of MDE within the vagina, a congenital epithelial lesion called vaginal adenosis [15, 16]. Vaginal adenocarcinomas (VACs) are believed to arise from vaginal adenosis because of the presence of adenosis lesions at the primary site of VACs [17]. Hence, better understanding in etiology of vaginal adenosis is crucial in order to develop preventive and therapeutic approaches for VACs. The etiology of vaginal adenosis and VAC is commonly associated with intrauterine exposure to estrogenic compounds, including diethylstilbestrol (DES) [18]. Women who were exposed to DES in the womb of mothers (DES daughters) developed vaginal adenosis [19] and had a ~40 times higher risk of developing a specific type of VAC, vaginal clear cell adenocarcinoma [20]. Developmental exposures of rodents to estrogenic compounds also induce vaginal adenosis [21–26].

In this study, we investigated the role of sine oculis homeobox homolog 1 (SIX1) in the cell fate commitment of VgE. The vertebrate *Six* genes are homologs of *Drosophila* ‘*sine oculis*’ (so) [27]. The *Six* genes comprise an evolutionally conserved gene regulatory network with paired box (*Pax*) and eyes absent (*Eya*) [28, 29]. In mammals, the *Six* genes (*Six1*–*6*) cooperatively regulate the developmental process in multiple organs [30, 31]. *Six1* null mutant mice die at birth, exhibiting craniofacial abnormalities and agenesis of thymus and kidney [32]. In humans, *SIX1* mutations cause Branchiootic syndrome 3 (MIM#608389) [33], characterized by hearing loss, branchial cleft fistulas/cysts and renal dysplasia. *SIX1* mutations also cause Deafness, autosomal dominant 23 (DFNA23, MIM#605192) [34], which is characterized by bilateral hearing impairment without renal malformations. In mouse FRTs, *Six1* is enriched in the vagina compared with the uterus [16, 35]. However, its biological function in FRT remains unclear.

Our current mouse genetic study reveals that SIX1 cooperates with RUNX1 and SMAD4 in the activation of the ΔNp63 locus in MDE as a downstream transcription factor of BMP4, ActA, and FGF7/10. Here, we provide evidence that DES blocks the activation of ΔNp63 locus in future VgE by repressing SIX1 and RUNX1 through epithelial estrogen receptor α (ESR1). Such discoveries from our models may contribute to developing preventive and therapeutic treatments of VACs, the etiology of which is currently unknown.

## Materials and methods

### Mouse models

All animal procedures were approved by the Animal Care and Use Committee in the Ohio State University. The mouse strains carrying the following alleles were utilized: *Six1*^*flox*^ [*Six1*^*tm2.1Mair*^] [36], *Trp63*^*flox*^ [*Trp63*^*tm3.2Brd*^] [37], ΔNp63-EGFP knock-in (*Trp63*^*ΔNp63-EGFP-KI*^) [38], *Runx1*^*flox*^ [*Runx1*^*tm1Tani*^] [39], *Fgfr2*^*flox*^ [*Fgfr2*^*tm1Dor*^*/J*] [40], *ROSA*^*mT-mE*^ [*Gt(ROSA)26Sor*^*tm4(ACTB-tdTomato,–EGFP)Luo*^*/J*] [41], *ROSA*^*MAP2K1DD*^ [*Gt(ROSA)26Sor*^tm8(Map2k1*,EGFP)Rsky^*/J*] [42], *Smad4*^*flox*^ (*Smad4*^*tm2.1Cxd*^*/J*) [43], *Esr1*^*flox*^ [44], *Pax2-Cre* [Tg(Pax2-cre)1Akg] [45], *Wnt7a-Cre* [46], and ΔNp63-Cre [*Trp63*^*tm1.1(cre)Ssig*^*/J*] [47]. C57BL/6J mice were purchased from Jackson Laboratory (Bar Harbor, ME). MDE-specific conditional knockout (cKO) and conditional heterozygous (cHET) mice were generated by crossing lines carrying floxed alleles with *Wnt7a-Cre* mice, except for *Trp63*^*flox*^ mice, which were crossed with *Pax2-Cre* because *Trp63*^*flox/flox*^; *Wnt7a-Cre* mice were embryonic lethal [16]. The day of birth was counted as postnatal day (PD) 1.

### Neonatal DES treatment

The ~0.04 mg/mm DES filled tubing [16] was cut into 1 mm lengths and subcutaneously injected into newborn mice using a 19-gauge trocar.

### Immunofluorescence (IF) and immunohistochemistry (IHC)

IF and IHC assays were performed as previously described [48]. The following primary antibodies were used at the indicated dilutions: anti-TRP63 (4A4) (1:200, 790–4509) from Ventana Medical Systems (Tucson, AZ); anti-ΔNp63 (1:2,000, PC373) from Millipore (Billerica, MA); anti-PGR (1:200, A0098) from Agilent Technologies (Santa Clara, CA); anti-RUNX1 (1:400, 2593-1) from Epitomics (Burlingame, CA); anti-phospho (p)-MAPK1/3 (p-T202/Y201, 1:30, #4370) and anti-pSMAD1/5/9 (1:50, #9511) from Cell Signaling Technology (Danvers, MA); anti-GFP (1:100, ab6673) from Abcam (Cambridge, MA); anti-SIX1 (1:800, HPA001893) from Millipore Sigma (St. Louis, MO); anti-ESR1 (1:100, RM-9101) from Lab Vision (Fremont, CA); anti-pan-Cytokeratin (AE1/AE3) (1:100, sc-81714) from Santa Cruz Biotechnology (Dallas, TX). For IF assay, Alexa-Fluor594 anti-mouse IgG (1:1,000, 715-586-150) and Alexa-Fluor488 anti-rabbit IgG (1:1,000, 711-546-152) (Jackson ImmunoResearch, West Grove, PA) were used for the secondary antibodies, and Hoechst 33258 (1:10,000, Sigma-Aldrich) was used for nuclear staining. For IHC with DAB (3,3′-diaminobenzidine, Sigma-Aldrich), biotinylated anti-rat goat IgG (1:800, 705-065-147) and streptavidin-horseradish peroxidase (1:400, 016-030-084) (Jackson ImmunoResearch) were used. Micrographs were captured using a BZ-9000 microscope (Keyence, Osaka, Japan) under identical conditions between samples for each antibody. The contrast of images was adjusted by applying identical parameters to the images for each antibody with the batch-process function of Adobe Photoshop CS6 (Adobe, San Jose, CA). To capture a wide area in a single image, tissue sections were scanned in multiple frames, and the images were automatically merged together utilizing the Image Stitching function of image analysis tool (Keyence).

### Morphometric analysis

The methods for the quantitative analysis on the squamous transformation of MDE [16] and the IF signal [49] were previously described. We adapted these methods with some modifications. The length of epithelium at the basal lamina was measured in the outer wall of vaginal fornix in at least two TRP63-immunostained coronal-sections per animal. The proportion of epithelium with ΔNp63-positive basal cells was calculated by “length of epithelial basement membrane associated with TRP63-positive cells” ÷ “total epithelial basement membrane length” × 100, for each mouse. Basal cell density in the outer and inner fornix walls was calculated by the number of TRP63-positive pixels per epithelial basement membrane length. In tissue sections of vaginal fornices stained for TRP63, epithelial areas were manually selected, and the pixels positive for TRP63 signal within the epithelium were selected by adjusting the lower threshold for positivity to exclude background noise. Epithelial basement membrane was manually marked on the IF images, and the p63-positive area and the basement membrane length were measured utilizing Image J (NIH, Bethesda, MD). Analysis was performed on ≥4 fornices from ≥3 mice per group. The value in each fornix was considered as a single measurement. Statistical significance was analyzed by One-way ANOVA with post-hoc Tukey’s honestly significant difference test.

### SIX1 IF analysis

Quantitative IF assay was performed as previously described with modifications [50]. Tissue sections were stained together, and images were captured at the same time under identical conditions. Images of ≥4 coronal tissue sections from *n* ≥ 3 independent animals were analyzed for each group. Epithelial areas were manually selected, and the signal intensity per pixel within the epithelial area was measured by Image J. In all experiments, approximately equivalent areas were analyzed in each sample. Since there was no significant intragroup difference in the average signal intensity, all samples in each group were plotted together, and the distributions of signals were compared between groups by the Mann–Whitney *U* test with continuity correction.

### Immunoblot analysis

Ovaries, uteri and vaginae from PD2 mice (5–6 mice per blot) were homogenized in ice-cold lysis buffer containing protease (cOmplete Protease Inhibitor Cocktail, Roche, Basel, Schweiz) and phosphatase (phoSTOP, Roche) inhibitors and loaded onto NuPAGE 4–12% Bis-Tris precast SDS-PAGE gel (ThermoFisher, Waltham, MA). Proteins were transferred to a PVDF membrane (Millipore Sigma). The membrane was incubated with anti-RUNX1 (1:2,000, Epitomics), anti-SIX1 (1:1,000, Millipore Sigma) and anti-GAPDH (1:2,000, G8795, Millipore Sigma) antibodies in the OdysseyR Blocking buffer (LI-COR Biosciences, NE) overnight at 4 °C. After incubation with IRDye^®^ 800CW and 680LT Donkey secondary antibodies, the signal was detected using Odyssey CLx Imaging System (LI-COR Biosciences). The analysis was repeated three times with independent samples.

### Uterine organ culture

Uterine hanging drop organ culture was performed as previously described with minor modifications [15, 16]. Briefly, uteri were dissected from PD1 mice, cleaned by removing connective tissues, and cut into 3 pieces per uterine-horn in Dulbecco’s Modified Eagle Medium/Nutrient Mixture F12 (DMEM/F12, 11039, ThermoFisher) containing 10 nM ICI 182,780 (Millipore Sigma). The uterine pieces were then placed in autoclaved PCR tube caps (AXYGEN, Union City, CA) with basal medium (10 nM ICI 182,780 DMEM/F12 with Insulin-Transferrin-Selenium and Antibiotic-Antimycotic) with/without 20 ng/ml human recombinant BMP4, ActA and/or FGF10 (ThermoFisher), inverted, and incubated. Uterine pieces were cultured up to 3 days with daily medium change, fixed with Modified Davidson’s fixative, and processed for histological analysis.

### Quantitative real-time PCR (qRT-PCR)

Newborn female mice were implanted with a vehicle (Veh, empty pellet) or DES pellet within 12 h after birth (*n* ≥ 3). At 24 and 48 h after pellet implantation, uterine horns and the upper half of vaginae were collected, immediately snap-frozen and stored at −80 °C until use. Total RNA was extracted from vagina and uteri of each mouse separately utilizing RNeasy Plus Mini or Micro Kits (Qiagen, Hilden, Germany). cDNA was synthesized using SuperScript II (ThermoFisher) with oligo(dT) primer, and qRT-PCR was performed on a CFX Connect Real-Time PCR Detection System (Bio-Rad, Hercules, CA) using KAPA SYBR FAST qPCR kits (Kapa Biosystems, Inc., Wilmington, MA). Primer sequences in this study are available upon request. The relative expression values of target transcripts were calculated by normalizing the threshold cycle (CT) value to that of *Cdh1* for epithelial genes (*Six1*, *Runx1*, *Fgfr2IIIb*, and *ΔNp63*), and *Vim* for mesenchymal genes (*Bmp4*, *Fgf7*, *Fgf10*, and *Inhba*). The average values of Veh-treated vaginal or uterine samples at 24 h were set as 1.0. The qRT-PCR data were statistically analyzed by *F*-test followed by a Student’s *t* test or Welch’s *t* test between Veh-treated and DES-treated tissues in each time point.

## Results

### Expression patterns of SIX1 in neonatal FRTs

**Fig. 1:**
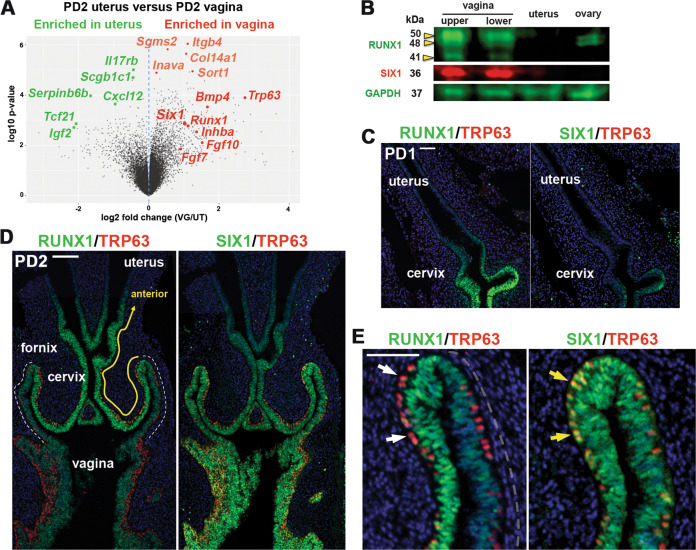
Expression patterns of SIX1 in developing female reproductive tract. **a** Volcano plot displaying differential expressed genes in mouse PD2 uterus and vagina. Genes significantly enriched in vagina and uterus in microarray analysis [16] are marked in red and green, respectively. **b** Immunoblot analysis of SIX1 and RUNX1 expression in PD2 mouse FRT and ovary. The vagina was divided into upper and lower half. Immunofluorescence assay for RUNX1, SIX1 (green) and TRP63 (red) in the lower FRT at PD1 (**c**) and PD2 (**d**, **e**). Outer wall of fornix is marker with dotted line (**d**). In the vaginal fornix (**e**), RUNX1 is downregulated in MDE upon expression of TRP63 (white arrows), whereas TRP63 and SIX1 are co-expressed (yellow arrows). Bar = 100 µm (**c**, **d**), =50 µm (**e**).

ΔNp63α is the dominant isoform of TRP63/TP63 in mouse/human VgE [14, 16]. To identify molecules that control epithelial cell fate in the lower FRT, we conducted microarray analysis of postnatal day 2 (PD2) vagina and uterus from MDE-specific cKO and conditional heterozygous (cHET, control) mice of *Trp63* with *Pax2-Cre* [16]. Differential gene expression between cHET vagina and cHET uterus (Fig. 1a) reflects both the upstream and downstream signaling of TRP63 [16], whereas the comparison of *Trp63* cKO and cHET vaginae identifies the downstream targets of TRP63. In the analysis, *Six1* was more enriched in vaginae than uteri (2.03-fold-change, *p* = 0.0013) (Fig. 1a). The level of *Six1* in the neonatal vagina was not significantly different between *Trp63* cKO and cHET mice (Log_2_ cHET/cKO = −0.176, *p* = 0.23) [16], indicating that *Six1* is not the target of TRP63. Immunoblotting confirmed the result: SIX1 protein was detected in PD2 vaginae but not in uteri (Fig. 1b).

SIX1 expression progressed from posterior to anterior in developing vagina. At birth, SIX1 was expressed in the MDE of the lower vagina but not in the upper vagina and cervix, where RUNX1 was already expressed (Fig. 1c). By PD2, SIX1 expression extended to the cervix (Fig. 1d). However, there were substantial differences in the expression patterns of RUNX1 and SIX1. RUNX1 expression was reduced in the posterior portion from the outer wall of the fornix (Fig. 1d, outer wall of fornix is marked with white dotted line), whereas SIX1 was expressed at similar levels in both inner and outer walls of the fornix (Fig. 1d). In addition, RUNX1 was downregulated upon expression of ΔNp63 (Fig. 1e, white arrow) [16], whereas SIX1 expression persisted in ΔNp63-positive cells (Fig. 1e, yellow arrow).

### Regulation of SIX1 expression in MDE

**Fig. 2:**
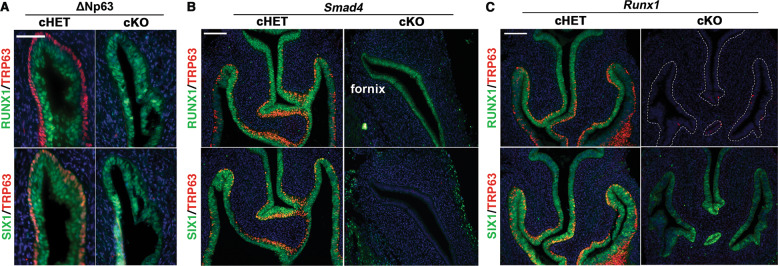
SIX1 is a downstream transcription factor of BMP4-SMAD pathway. In all figures, outer wall of fornix is shown on the right side. **a** SIX1 expression is maintained in the vaginal fornix of ΔNp63 cKO mice (PD14) (*n* ≥ 4). Bar = 50 µm. **b** SIX1 expression in MDE is SMAD4 dependent. At PD2, SIX1 is totally absent in the MDE of *Smad4* cKO mice, which normally express RUNX1 in MDE. **c** Expression of RUNX1 and SIX1 in the lower FRT of PD2 *Runx1* cHET and cKO mice. RUNX1 null vaginal/cervical epithelium is outlined by doted lines. Nuclear expression of SIX1 expression is reduced in the fornices of *Runx1* cKO mice. Bars = 100 µm.

**Fig. 3:**
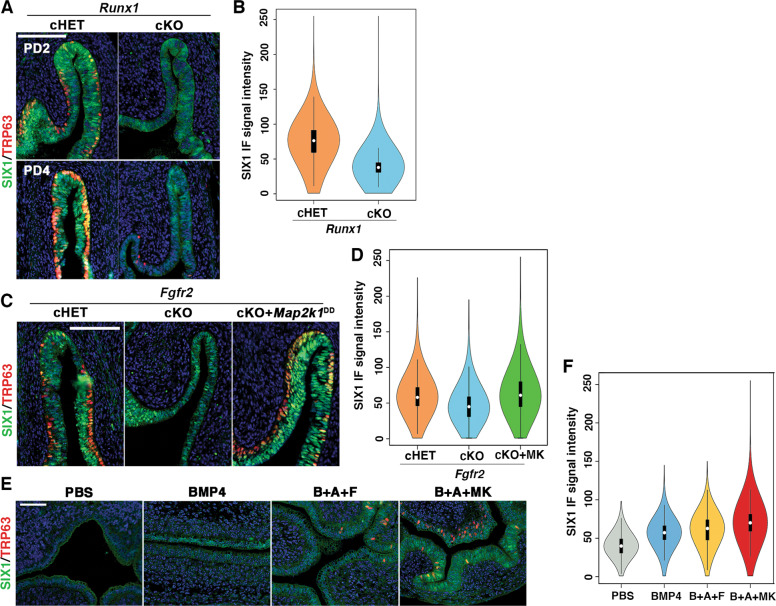
RUNX1 and FGFR2 modulate expression levels of SIX1 in MDE. **a** SIX1 expression patterns in the vaginal fornices of *Runx1* cHET and cKO mice at PD2 and PD4. In the fornix of *Runx1* cKO mice, nuclear expression of SIX1 increases from PD2 to PD4, but the overall expression level of SIX1 in MDE remains low and uneven. **b** Violin plot of SIX1 IF signal distribution in the fornix of PD2 *Runx1* cHET and cKO mice (*n* ≥ 4 per group). The signal distributions of two groups are significantly different (*p* < 2 × 10^–16^). **c** Expression of SIX1 in the vaginal fornix of *Fgfr2* mutant mice. SIX1 is reduced in the fornix of *Fgfr2* cKO mice, but the SIX1 expression level is restored by expression of MAP2K1^DD^. **d** Violin plot of SIX1 IF signal distribution in the fornix of PD2 *Fgfr2* cHET, *Fgfr2* cKO, and *Fgfr2* cKO with MAP2K1^DD^ (cKO + MK) mice (*n* = 4 per group). The signal distributions are significantly different among three groups (*p* < 2 × 10^–16^). **e** Regulation of SIX1 in cultured uterine explants. 20 ng/ml BMP4 has a weak effect on the expression of SIX1 in UtE. The combination of BMP4 (**b**), ActA (**a**), and FGF10 (**f**) (20 ng/ml each) induced nuclear expression of SIX1 and ΔNp63 in restricted regions of UtE. Replacement of FGF10 with *Map2k1*^DD^ transgene (MK) efficiently induced SIX1 and ΔNp63 in UtE. **f** Violin plot of SIX1 IF signal distribution in the UtE of cultured uterine explants (*n* ≥ 4 per group). The signal distributions are significantly different among groups (*p* < 2 × 10^–16^). Bars = 100 µm.

SIX1 was expressed in the fornices of ΔNp63 cKO and cHET mice [16] at PD14, confirming that expression of SIX1 is independent of ΔNp63 (Fig. 2a). In contrast, PD2 *Smad4* cKO mice with *Wnt7a-Cre* [16] completely lacked the expression of SIX1 in the entire MDE (*n* = 5) (Fig. 2b). In PD2 *Runx1* cKO mice with *Wnt7a-Cre* [16], SIX1 was expressed throughout the VgE, however the expression of SIX1 in the fornix was reduced compared with *Runx1* cHET mice (Figs. 2c and 3a, b; *p* value < 2.2 × 10^–16^). This was not due to delayed development, as SIX1 expression in the vaginal fornix remained low in PD4 *Runx1* cKO mice (Fig. 3a). Thus, the expression level of SIX1 in MDE is positively regulated by ActA-RUNX1 signaling activity. Similarly, SIX1 expression was slightly reduced in the fornix of *Fgfr2* cKO mice with *Wnt7a-Cre*, in which vaginal MDE undergoes uterine differentiation [15] (Fig. 3c). However, SIX1 expression in the fornix was upregulated when the vaginal defect of *Fgfr2* cKO MDE was corrected with the expression of a constitutively active MAP2K1 (MAP2K1^DD^) [15] (Fig. 3c, d, cHET v.s. cKO; *p* value < 2 × 10^–16^, cKO v.s. cKO + MK; *p* value < 2 × 10^–16^, cHET v.s cKO + MK; *p* value < 2 × 10^–16^), suggesting that MAPK activity modulates the expression level of SIX1 in the vaginal fornix. Accordingly, we tested the effect of BMP4, ActA and FGF10, three vaginal mesenchymal factors that induce ΔNp63 in MDE, on SIX1 expression in uterine organ culture assay. ActA and FGF10 had no effect on SIX1 expression in uterine explants and only BMP4 slightly increased SIX1 in UtE, but nuclear expression was mostly absent (Fig. 3e). When all three factors were combined, nuclear SIX1 expression was detected in a portion of UtE. The areas showing nuclear SIX1 expression also contained ΔNp63-positive cells, suggesting that SIX1 is involved in the signaling pathway that activates ΔNp63 in MDE. In uterine organ culture, diffusion of FGF10 within connective tissues is limited, due to its high affinity to heparan sulfate [51]. Accordingly, we replaced FGF10 with the epithelial expression of MAP2K1^DD^, which by itself did not induce expression of ΔNp63 [15]. ActA and BMP4 efficiently induced SIX1 as well as ΔNp63 in *Map2k1*^*DD*^ transgenic UtE (Fig. 3e, f), suggesting that SIX1 is the downstream transcription factor of vaginal mesenchymal factors.

### *Six1* and *Runx1* dose-dependently promote ΔNp63 expression in MDE

**Fig. 4:**
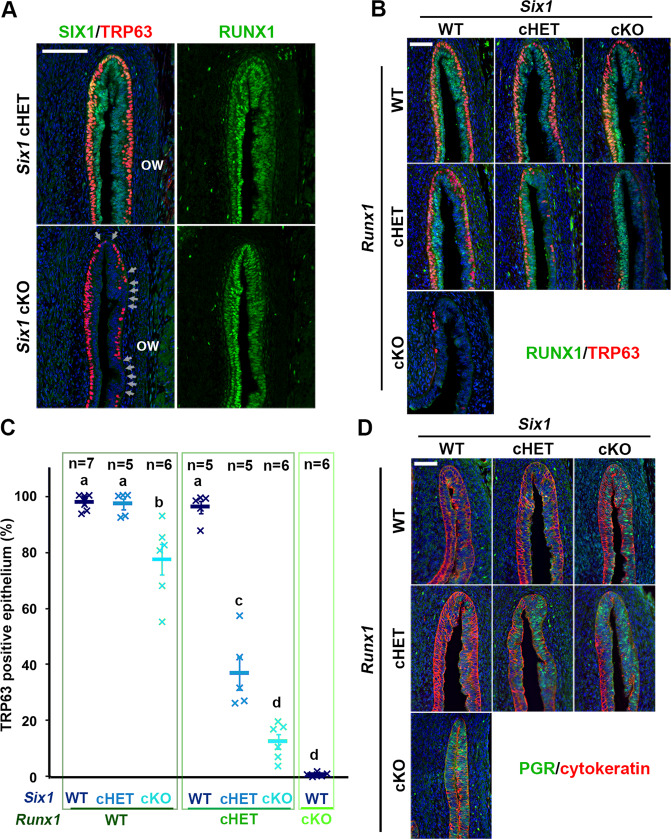
SIX1 and RUNX1 collaborate in the activation of ΔNp63 locus in MDE. **a** *Six1* cKO mice showed minor defects in ΔNp63 expression in the outer wall (ow) of vaginal fornix. The ΔNp63-negative epithelial regions are indicated by arrows. **b**–**d** Gene-does effect of *Six1* and *Runx1* on vaginal cell fate commitment of MDE in the vaginal fornix. The outer fornix wall is on the right side. **b** Expression of ΔNp63 (red) and RUNX1 (green). **c** Proportion of MDE lined with ΔNp63-psotive basal layer on the outer wall of vaginal fornix. **d** Expression of uterine epithelial marker (PGR, green). The epithelium is highlighted with cytokeratin (red). Bars = 100 µm.

Since *Six1* null mice die before vaginal epithelial differentiation occurs [52], the role of SIX1 in VgE differentiation was assessed by genetically inactivating *Six1* in MDE by *Wnt7a-Cre* [46]. The loss of SIX1 in MDE affected the formation of the ΔNp63-positive basal epithelial layer in the vaginal fornix (Fig. 4a). Thus, SIX1 is one of several key transcription factors in the vaginal cell fate commitment of MDE. However, the vaginal defect of *Six1* cKO mice was relatively minor compared with *Smad4*, *Runx1*, and *Fgfr2* cKO mice: while *Smad4*, *Runx1*, and *Fgfr2* cKO mice lost ΔNp63 expression in a significant portion of vagina, the defect of *Six1* cKO mice was restricted to the epithelium on the outer wall of vaginal fornix, where the expression of RUNX1 is reduced (Fig. 1d). Meanwhile, RUNX1 expression in the vaginal fornix was not affected in *Six1* cKO mice (Fig. 4a). Hence, we generated compound conditional mutant mice of *Six1* and *Runx1* with *Wnt7a-Cre* to assess if SIX1 and RUNX1 collaborate in the ΔNp63 expression of MDE in the outer wall of the vaginal fornix. Monoallelic loss of *Runx1* in MDE exaggerated the effect of *Six1* allelic loss on ΔNp63 expression: While monoallelic loss (cHET) of *Six1* or *Runx1* alone had no evident effect on the formation of ΔNp63-positive basal layer, the *Six1;Runx1* double cHET mice showed gaps in the ΔNp63-positive basal cells in the outer wall of the fornix (Fig. 4b, c). The ΔNp63-negative epithelial area expanded further to the inner wall of the fornix when biallelic loss (cKO) of *Six1* was combined with monoallelic loss of *Runx1* (Fig. 4b, c). Moreover, the ΔNp63-negative MDE within the fornix expressed progesterone receptor (PGR), indicating the uterine cell fate commitment [53] (Fig. 4d).

### Gene-dose-dependent function of *Six1*, *Runx1*, and *Smad4* in activation of ΔNp63 locus in MDE

**Fig. 5:**
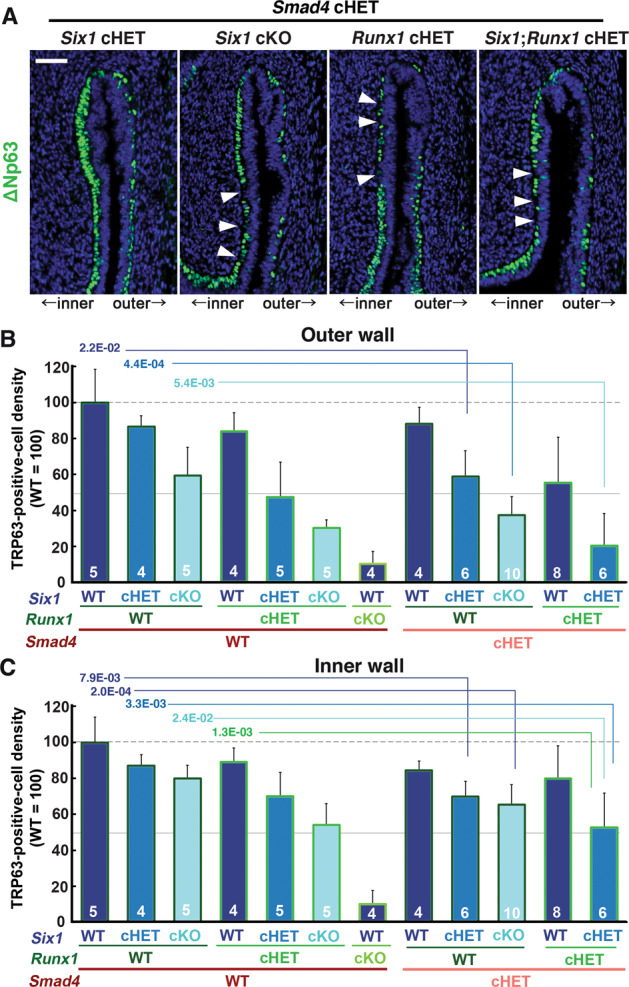
Dose-dependent function of *Six1*, *Runx1* and *Smad4* in the activation of ΔNp63 locus. **a** Monoallelic loss of *Smad4* exaggerates effects of *Six1* and *Runx1* null alleles on ΔNp63 expression (green) in MDE. The outer fornix wall is shown on the right side. Breaks in the ΔNp63-positive basal layer in the inner fornix wall are marked by arrowheads. Bar = 50 µm. **b**, **c** Basal cell density (TRP63-positive nuclear area per epithelial basement membrane length) in the outer and inner fornix walls of *Six1*, *Runx1*, and *Smad4* compound mutant mice. The sample number in each group is marker on the bars. The result is demonstrated by average means ± SD. The comparisons that become significantly different by monoallelic *Smad4* loss are marked by lines with *p* value.

The distinctive vaginal phenotypes of *Six1* cKO and *Smad4* cKO mice indicate that SMAD4 works independent of SIX1 in vaginal cell fate commitment of MDE. Accordingly, we assessed if the efficacy of SIX1 and RUNX1 in the activation of ΔNp63 expression in MDE is affected by monoallelic loss of *Smad4* gene, which alone does not affect the formation of ΔNp63-positive basal layer in VgE [16]. *Six1;Smad4* double cHET mice expressed ΔNp63 throughout the vagina at PD4. However, the density of basal cells on the outer wall of the fornix was reduced (Fig. 5a). The combinatorial effect of *Six1* and *Smad4* alleles became more prominent when an additional *Six1* allele was inactivated (Fig. 5a). Similarly, monoallelic loss of *Smad4* and *Runx1* does-dependently affected the density of ΔNp63 in the fornix (Fig. 5a). Accordingly, *Six1;Smad4;Runx1* triple cHET mice demonstrated gaps in the ΔNp63-positive basal layer throughout the vaginal fornix (Fig. 5a). The effect of monoallelic *Smad4* loss on the density of TRP63-positive cells was statistically significant in mice with certain genotypes (Fig. 5b, c, Table S1). For instance, the TRP63-positive cell density in *Six1* cHET mice became significantly lower than WT mice with the monoallelic loss of *Smad4* (*Six1* cHET; *Smad4* cHET) (Fig. 5b, c).

### Regulatory elements of ΔNp63

The gene-dose-dependent effect of *Six1, Runx1*, and *Smad4* on ΔNp63 activation suggests collaboration among these transcription factors. The analysis of evolutionally conserved regions by ECR browser [54] identified numerous numbers of putative enhancer elements within *TP63*/*Trp63* locus. Many of these conserved sequences near ΔNp63 TSS contained binding sites for SMADs, RUNX1 and SIX1 (Fig. S1). The 5′ sequence proximal to ΔNp63 TSS, to which SMAD4 binds in VgE [16], also contained binding sites of RUNX1 and SIX1 (Fig. S2A). Thus, we generated transgenic mice to test if the putative 5′ proximal enhancer and the promoter are sufficient to replicate the expression patterns of ΔNp63 (Fig. S2). However, the transgene (Cre-ires-EGFP) was not expressed in any tissues of five founders and their progenies, indicating the insufficiency of the sequence to replicate ΔNp63 expression. Furthermore, when ΔNp63-Cre knock-in mice, in which the coding sequence in the first exon of ΔNp63 was replaced with Cre [47] were crossed with *ROSA*^*mT-mE*^ reporter mice [41], VgE was mostly negative for mEGFP (*n* = 3, Fig. S2C). ConTra v3 analysis [55] identified conserved binding sites of SMAD1, SMAD4 and RUNX1 within the sequence deleted in ΔNp63-Cre knock-in mice (Fig. S1E). Thus, the efficient activation of ΔNp63 locus in MDE appears to require cooperation of multiple regulatory elements including the protein coding sequence within exon 1.

### Developmental exposure to diethylstilbestrol (DES) downregulates *Runx1* and *Six1* in the vaginal fornix

**Fig. 6:**
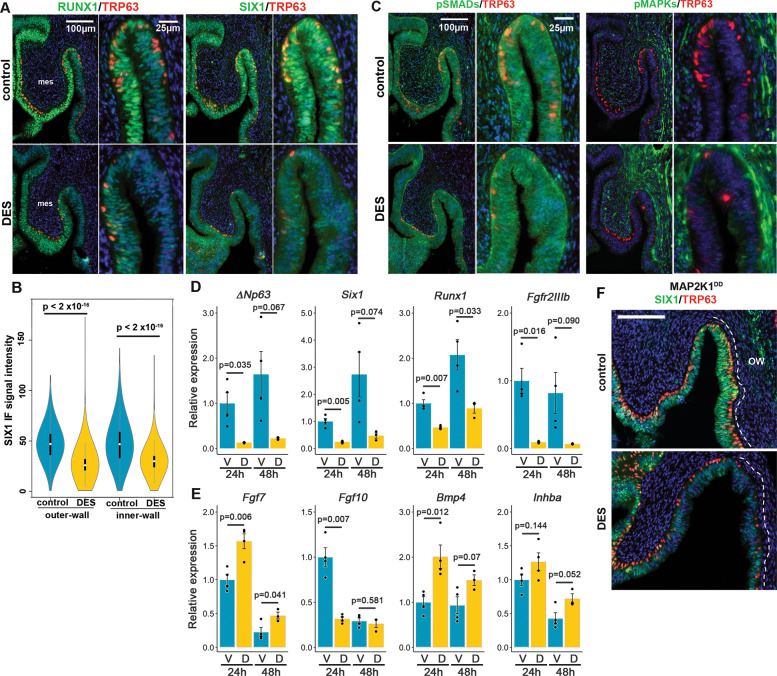
DES inhibits expression of SIX1 and RUNX1 in the vaginal fornix. IF assay of RUNX1, SIX1 (**a**), pSMAD1/5/9 and pMAPK1/3 (**b**) Violin plot presentation of SIX1 IF signals in the outer and inner fornix walls of control and DES-treated PD2 mice (*n* = 4 each). SIX1 IF signals in MDE were significantly higher (***p < 2 × 10^–16^) in control than DES-treated mice in both outer and inner fornix walls. **c** for DES effects in MDE. mes; mesenchyme. FRTs are collected from PD2 female mice with/without DES treatment (24 h after DES-pellet injection). **d**, **e** Effect of DES on transcript levels of essential factors for ΔNp63 expression in the upper part of vagina. Epithelial genes (**d**) are normalized by *Cdh1*. Mesenchymal genes (**e**) are normalized by *Vim*. V, Vehicle; D, DES. **f** Effect of DES on the SIX1 (green) and TRP63 (red) expression in the fornix of *Map2k1*^*DD*^ transgene mice. Bars = 100 µm.

Previously, we demonstrated that downregulation of RUNX1 is involved in the pathogenesis of DES-associated vaginal adenosis [16] (Fig. 6a). However, the effect of 24-h DES treatment was more prominent on SIX1 than RUNX1: Nuclear expression of SIX1 disappeared from the MDE in the vaginal fornix and the cervix of DES-treated mice (Fig. 6b, c). Meanwhile, the downregulation of SIX1 was not likely due to the repression of BMP4-SMAD or FGF7/10-MAPK activities because DES treatment increased pMAPK1/3 in VgE and mesenchyme while it had no evident effect on the levels of pSMAD1/5/9 (Fig. 6c).

We tested the effect of DES on mRNA levels of factors essential for ΔNp63 expression. Consistent to IF analysis, DES reduced the transcript level of *Six1* in the upper vagina by 76% at 24 h and 83% at 48 h compared with the vehicle (Veh) control (Fig. 6d). *Runx1* mRNA was also downregulated by ~50% in DES-treated group (Fig. 6d). In contrast, DES significantly upregulated transcripts for *Fgf7* and *Bmp4* in the upper vagina at 24 and 48 h (Fig. 6e). *Inhba*, encoding subunit of ActA, was also upregulated, but it did not reach statistically significant levels (Fig. 6e). Among four vaginal mesenchymal factors we measured, only *Fgf10* was downregulated in the DES group at 24 h. Nevertheless, *Fgf10* mRNA levels became comparable between DES and control groups by 48 h (Fig. 6e). Unexpectedly, DES reduced the expression of *Fgfr2IIIb* by more than 80% (Fig. 6e), suggesting a possibility that DES alters the epithelial cell fate in the upper vagina by blocking FGF7/10-MAPK pathway via downregulation of *Fgfr2IIIb*. However, DES increased the phosphorylation of MAPK1/3 in MDE within the fornix (Fig. 6c). Furthermore, nuclear staining of SIX1, which was evident in the MDE of *Fgfr2* cKO mice (Fig. 3c), disappeared from the outer fornix of DES-treated mice (Fig. 6a). These observations do not support the hypothesis that DES downregulates SIX1 by blocking the FGF-MAPK pathway. Thus, we tested if the activation of MAPK1/3 by *Map2k1*^*DD*^ counteracts DES effects on ΔNp63 and SIX1 expression in MDE. DES repressed the expression of SIX1 and ΔNp63 in *Map2k1*^*DD*^ conditional transgenic mice with *Wnt7a-Cre* (Fig. 6f), rejecting the hypothesis.

Interestingly, DES upregulated mRNAs for vaginal mesenchymal factors and downstream transcription factors in the uterus (Fig. S3A). In the mouse uterus, ESR1 is expressed in the mesenchyme but not in the epithelium at birth [7, 56, 57], suggesting that DES induces vaginal mesenchymal factors in the uterus via mesenchymal ESR1.

### DES inhibits activation of ΔNp63 locus in MDE through downregulation of RUNX1 and SIX1 via epithelial ESR1

**Fig. 7:**
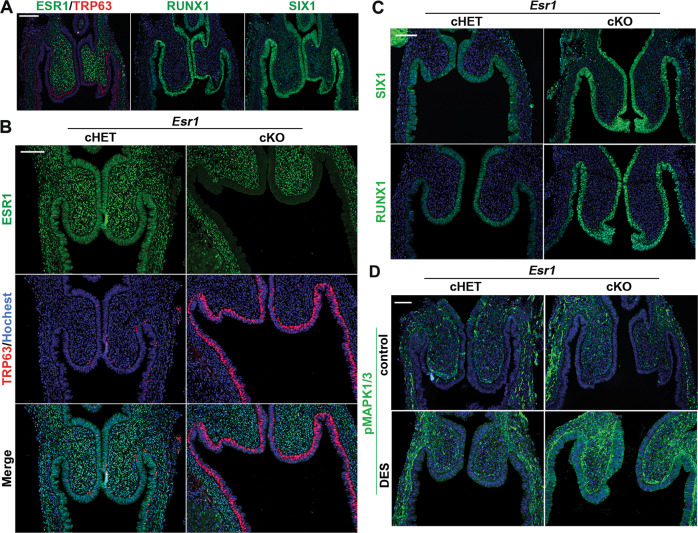
Epithelial ESR1 mediates DES effects on ΔNp63 in developing vagina. **a** Expression patterns of ESR1 (green) and TRP63 (red) in *Esr1* cKO mice (PD3). RUNX1 and SIX1 in *Esr1* cKO mice are indistinguishable from wild type mice. Effect of DES on the FRT of *Esr1* cHET and cKO mice (PD3): (**b**) IF assay of ESR1 (green) and TRP63 (red), (**c**) IF assay of SIX1 and RUNX1. **d** Effect of DES (or control) on the phosphorylation of MAPK1/3 (green). Bars = 100 µm.

Our previous tissue recombination study has established that DES blocks expression of ΔNp63 in MDE through ESR1 within the epithelial cells [12, 58]. In the experiment, expression of ΔNp63 in ESR1 null MDE was not inhibited by direct contact with ESR1-positive MDE, reasonably excluding the involvement of a juxtacrine or paracrine mechanism among epithelial cells [58]. However, the fornix-specific effect of DES was not assessed in these studies, as the anatomical structure of vagina was lost by tissue recombination. We generated MDE-specific *Esr1* cKO mice with *Wnt7a*-*Cre*. The deletion of *Esr1* in MDE did not change the expression patterns of SIX1, RUNX1 and ΔNp63 in the fornix (Fig. 7a). DES did not block the induction of ΔNp63 in the VgE of *Esr1* cKO mice (Fig. 7b). Instead, DES exposure promoted the expression of ΔNp63 in VgE in *Esr1* cKO mice (Fig. 7b), forming a continuous layer of ΔNp63-positive cells by PD3, ≥1 day earlier than normal development. This is likely due to the up-regulation of BMP4 and ActA (Fig. 6e). DES induced RUNX1 and SIX1 in the UtE of *Esr1* cKO mice (Fig. S3B), further demonstrating that DES action via mesenchymal ESR1 promotes vaginal epithelial cell fate in MDE. The expression of SIX1 and RUNX1 was maintained in the vaginal fornices of DES-treated *Esr1* cKO mice (Fig. 7c), suggesting that DES-ESR1 attenuates the expression of SIX1 and RUNX1 in MDE cell autonomously.

Interestingly, DES increased MAPK1/3 activities in the entire vagina of *Esr1* cKO mice (Fig. 7d). This suggests that DES activates the MAPK1/3 pathway in MDE and vaginal mesenchymal via ESR1 in the mesenchyme.

## Discussion

It has long been known that the differentiation of MDE into distinctive epithelia of uterus and vagina is under the control of organ-specific mesenchyme [9]. Our group has established that ΔNp63 is the master regulator of vaginal epithelial differentiation in MDE [12], and that the expression of ΔNp63 is induced by mesenchymal paracrine factors, BMP4, ActA, and FGF7/10 [15, 16]. Within MDE, the signals from underlying mesenchyme are transduced by BMP4-SMADs, ActA-RUNX1, and FGFs-MAPKs. Since mouse vaginal mesenchyme can induce ΔNp63 and squamous differentiation in human MDE, the molecules that mediate communication between mesenchyme and epithelium in the commitment of MDE to vaginal cell fate must be common between these two species [59].

**Fig. 8:**
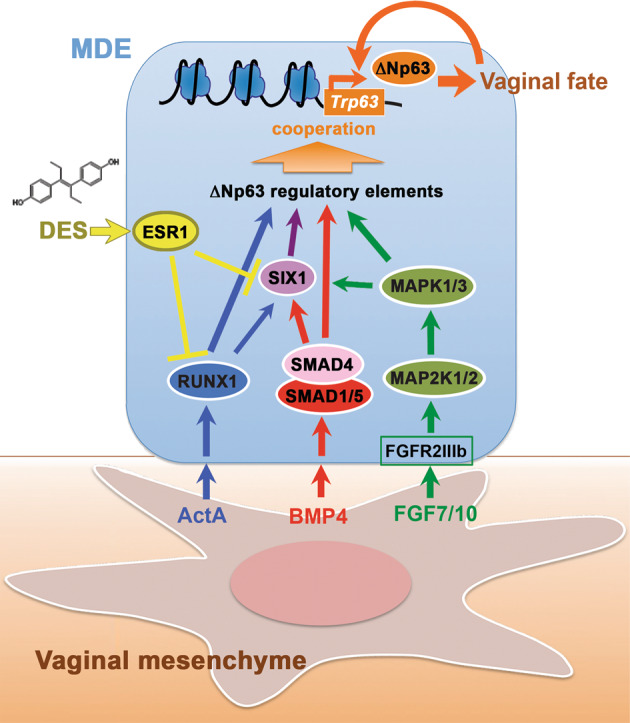
Model of vaginal epithelial cell fate commitment in MDE. Signals of vaginal mesenchymal factors are transduced to downstream transcription factors, and the transcription factors dose-dependently activate enhancers of ΔNp63 in MDE. Upon differentiation of VgE, ΔNp63 itself maintains the transcriptional activity of ΔNp63 locus in VgE fate independently of vaginal mesenchymal factors. DES-ESR1 activity within MDE causes vaginal adenosis by blocking the vaginal cell fate commitment of MDE interfering the signal transduction.

In this study, we identified SIX1 as one of several key transcription factors that mediate the mesenchymal signals in the activation of ΔNp63 locus during vaginal cell fate commitment of MDE. Subsequently, we propose that vaginal mesenchymal factors induce MDE to commit to vaginal epithelial cell fate by activating the ΔNp63 locus through cooperation of multiple enhancer elements, which are activated by SMADs, RUNX1, and/or SIX1 (Fig. 8). An enhancer is a short genomic region that contains clustered binding sites for multiple transcription factors. Although many transcription factors cannot bind their target site in the context of nucleosomal DNA, enhancer-mediated simultaneous binding of multiple transcription factors can overcome the nucleosome barrier [60]. Thus, enhancers integrate multiple signaling pathways through binding of downstream effectors, and regulate gene expression by organizing accessible chromatin in cooperation with promoters [61, 62]. In cell fate commitment of MDE to VgE, BMP, ActA, and FGF pathways are integrated to prime MDE for VgE-specific gene expression programs through the simultaneous binding of SMADs, RUNX1, and SIX1 to ΔNp63 enhancers (Fig. 8). The enhancers that regulate ΔNp63 expression in MDE must be distinctive from those in the skin because *Six1* null [52] and *Runx1* null [63] mice do not exhibit the deformation of skin and appendages observed in ΔNp63 mutant mice [38]. The identification of key regulator elements of ΔNp63 in MDE is imperative to fully appreciate the pathogenesis of vaginal adenosis. The usage of ΔNp63 enhancers must be unique between different regions of MDE as demonstrated by the difference in the requirement of SMAD4, RUNX1, and SIX1 for ΔNp63 expression in mouse genetic studies. Our particular interest is in the ΔNp63 enhancers utilized by MDE in the outer wall of the vaginal fornix, the primary site of vaginal adenosis development. Given the heterogeneity of the cell population (only a subpopulation of epithelial cells in the vaginal fornix express ΔNp63), the narrow developmental time window (ΔNp63 expression in the upper vagina gradually progresses from caudal to cranial between PD1 and PD4), and the small tissue amount of MDE within the fornix of neonatal mice, the identification of ΔNp63 regulatory elements in MDE by current technologies is challenging.

Since the expression patterns of ΔNp63 and RUNX1 as well as the effect of DES on the expression of these transcription factors are identical between human and mouse MDE [1, 14, 59, 64], the molecular model established in mice (Fig. 8) should explain the etiology of vaginal adenosis in DES daughters. However, VACs also occur in women who have no history of DES exposure [18, 65]. In human fetuses, the expression of ΔNp63 in the lower MD occurs during the first trimester [14, 64]. Hence, the pathogenesis of non-DES-associated VACs should involve an in utero event that disturbs cell fate commitment in MDE. In this regard, exposure to a compound that inhibits any pathways/molecules described in Fig. 8 can lead to vaginal adenosis. Some studies suggest the de novo formation of adenosis in the vagina of adult women following intravaginal applications of 5-fluorouracil cream [66–68], challenging our hypothesis. However, given the low detection sensitivity of routine cytology screenings for adenosis [69–71], adenosis cases that reported to be de novo are probably due to an increased visibility of previously imperceptible adenosis lesions enlarged by a reactive change to medical treatments.

In addition to vaginal adenosis, perinatal DES exposure of female mice induces uterine squamous metaplasia [72], a formation of squamous epithelium within the UtE. The gene expression pattern suggests that uterine squamous metaplasia results from vaginal cell fate commitment of MDE within the uterus [12, 58]. This intriguing dual-effect of DES is explained by the opposite functions of epithelial versus mesenchymal ESR1: DES action through epithelial ESR1 interferes the activation of ΔNp63 locus, whereas DES action through mesenchymal ESR1 promotes ΔNp63 expression (Fig. S4). When ESR1 is expressed in both epithelium and mesenchyme, DES effects via epithelial ESR1 are dominant. The molecular mechanisms through which DES-ESR1 represses RUNX1 and SIX1 in MDE remain unclear. Further study to elucidate the underlying molecular pathogenesis of DES-associated adenosis is essential to identify etiology of non-DES-associated vaginal adenosis and VAC.

## Supplementary information

Figure S1

Figure S2

Figure S3

Figure S4

Table S1
